# *Coriandrum sativum* L. (Coriander) Essential Oil: Antifungal Activity and Mode of Action on *Candida* spp., and Molecular Targets Affected in Human Whole-Genome Expression

**DOI:** 10.1371/journal.pone.0099086

**Published:** 2014-06-05

**Authors:** Irlan de Almeida Freires, Ramiro Mendonça Murata, Vivian Fernandes Furletti, Adilson Sartoratto, Severino Matias de Alencar, Glyn Mara Figueira, Janaina Aparecida de Oliveira Rodrigues, Marta Cristina Teixeira Duarte, Pedro Luiz Rosalen

**Affiliations:** 1 Pharmacology, Anesthesiology and Therapeutics, Department of Physiological Sciences, Piracicaba Dental School, State University of Campinas, Piracicaba, SP, Brazil; 2 Division of Periodontology, Diagnostic Sciences, and Dental Hygiene and the Division of Biomedical Sciences, Ostrow School of Dentistry, University of Southern California, Los Angeles, California, United States of America; 3 Research Center for Chemistry, Biology and Agriculture, State University of Campinas, Campinas, SP, Brazil; 4 Department of Agri-food Industry, Food and Nutrition, “Luiz de Queiroz” College of Agriculture, University of São Paulo, Piracicaba, SP, Brazil; 5 Postgraduate Program of Inter-units in Bioengineering, School of Engineering of São Carlos, University of São Paulo, São Carlos, SP, Brazil; Warren Alpert Medical School of Brown University, United States of America

## Abstract

Oral candidiasis is an opportunistic fungal infection of the oral cavity with increasingly worldwide prevalence and incidence rates. Novel specifically-targeted strategies to manage this ailment have been proposed using essential oils (EO) known to have antifungal properties. In this study, we aim to investigate the antifungal activity and mode of action of the EO from *Coriandrum sativum* L. (coriander) leaves on *Candida* spp. In addition, we detected the molecular targets affected in whole-genome expression in human cells. The EO phytochemical profile indicates monoterpenes and sesquiterpenes as major components, which are likely to negatively impact the viability of yeast cells. There seems to be a synergistic activity of the EO chemical compounds as their isolation into fractions led to a decreased antimicrobial effect. *C. sativum* EO may bind to membrane ergosterol, increasing ionic permeability and causing membrane damage leading to cell death, but it does not act on cell wall biosynthesis-related pathways. This mode of action is illustrated by photomicrographs showing disruption in biofilm integrity caused by the EO at varied concentrations. The EO also inhibited *Candida* biofilm adherence to a polystyrene substrate at low concentrations, and decreased the proteolytic activity of *Candida albicans* at minimum inhibitory concentration. Finally, the EO and its selected active fraction had low cytotoxicity on human cells, with putative mechanisms affecting gene expression in pathways involving chemokines and MAP-kinase (proliferation/apoptosis), as well as adhesion proteins. These findings highlight the potential antifungal activity of the EO from *C. sativum* leaves and suggest avenues for future translational toxicological research.

## Introduction

Oral candidiasis is a common opportunistic fungal infection of the oral cavity with worldwide prevalence and incidence rates increasing in the last several decades, particularly among the populations of HIV-immunocompromised and hospitalized individuals [1]. It is estimated that *Candida albicans* accounts for over 42% of fungal infections worldwide, followed by non-*albicans* species such as *C. glabrata, C. parapsilosis, C. tropicalis, C. krusei, C. guilliermondii, C. rugosa* and *C. dubliniensis* [2], [3].

Overall, the number of synthetic antifungal compounds and classes of therapeutic agents available to treat candidiasis has been increasing in recent decades, including polyenes, azoles, purine analogues and echinocandins [4]. As fungal pathogens are eukaryotes, however, they share some of their biological processes with human cells, which leads to major harmful side effects from the use of antifungal drugs [5]. Furthermore, the increased resistance of pathogens to synthetic agents [6], [7] and the need for cost-effective treatments to manage oral candidiasis have driven the search for novel alternatives in this field.

With this perspective, naturally-occurring agents stand out as a source of bioactive molecules with potential therapeutic application in the medical and dental fields [8]. Among these, essential oils (EO) are considered highly promising groups of natural compounds in terms of prevention and treatment of fungal infections [9].

One species with a potential antifungal activity is *Coriandrum sativum* L., popularly known as coriander. It is a small annual plant belonging to the Apiaceae family in the order of Apiales which originating from the Eastern Mediterranean where its use dates back to around 1,550 BC [10]. Coriander leaves and seeds are widely used in folk medicine as a cholesterol-lowering agent, a digestive stimulant, and an anti-hypertensive agent [11], in addition to its use as a seasoning in food preparation. Pharmaceutical applications of *C. sativum* have also revealed antibacterial [12], antioxidant [13], hepatoprotective [14] and anticonvulsant [15] activities.

The EO from *C. sativum* has been proven to have a strong antifungal effect against *Candida* species [9], [16], [17]. However, most studies have analyzed the EO from fruits [17] and seeds [9], which have a different chemical composition from those present in the leaves [16], [18]. Considering that *C. sativum* warrants further investigation, e.g. for the treatment of denture-related oral candidiasis, there is a need to elucidate the antifungal activity of the EO from its leaves with regard to mode of action, activity against *Candida* biofilms, and to perform pharmacogenomic analyses for toxicological purposes.

Thus, the present study aims to investigate the antifungal activity and mode of action of the EO from *Coriandrum sativum* L. (coriander) leaves on clinically relevant *Candida* species, and to determine the molecular targets affected through a whole-genome expression analysis in human cells.

## Materials and Methods

### Plant Material

*Coriandrum sativum* L. was obtained from the germoplasm bank of the Collection of Medicinal and Aromatic Plants (CPMA) at the Research Center for Chemistry, Biology and Agriculture (CPQBA), University of Campinas (UNICAMP), SP, Brazil (available from: http://webdrm.cpqba.unicamp.br/cpma/), and identified by G. M. Figueira (CPMA curator).

The plant material was collected between November 2012 and January 2013, during the morning after the dew-point. A voucher specimen was deposited in the herbarium of the Institute of Biology at UNICAMP (Campinas, SP, Brazil) and also registered in the herbarium of CPQBA, receiving an identification number (CPMA voucher # 644).

### Essential Oil Extraction

The EO was obtained using 100 gm of the leaves processed in several batches by hydrodistillation for 3 hours in a Clevenger-type system. The aqueous phase was extracted three times with 50 ml of dichloromethane. The organic layer was then isolated, dried with anhydrous sodium sulphate (Na_2_SO_4_), and filtered; the solvent was evaporated to obtain the crude EO. The oil content was stored at −20°C in amber sealed glass vials. Stock dilutions of the EO were prepared using 6.25% propylene glycol (v/v) as vehicle.

A previous study [16] fractionated the EO from *C. sativum* leaves using the dry column fractionation method and identified 10 fractions by thin layer chromatography which were grouped into 5 fractions based on chemical similarity (F_3_–F_4_, F_5_, F_6_, F_7_, F_8_–F_10_). These fractions were tested against *Candida* clinical isolates and culture collection strains, and the fraction showing the lowest Minimum Inhibitory and Fungicidal Concentration (MIC/MFC) values was considered to be the “active” one *(F_8_*–*F_10_)*. In our study, we extracted the EO and performed the fractionation process [16] to obtain this “active fraction” *(F_8_*–*F_10_)* to further microbiological and pharmacogenomic testing.

### Phytochemical Analysis by Gas Chromatography Coupled to Mass Spectrometry (GC-MS)

The profile of volatile constituents was determined using a Hewlett-Packard 6890 gas chromatograph coupled with an HP-5975 mass selective detector and HP-5 capillary column (30 m×0.25 mm×0.25 µm diameter). GC-MS analysis was performed using split injection (40∶1), with the injector set at 220°C, column set at 60°C, with a heating ramp of 3°C/min and a final temperature of 240°C, and the MS detector set at 250°C. Helium was used as a carrier gas at 1 ml/min. The GC-MS electron ionization system was set at 70 eV. Samples of the EO and active fraction were solubilized in ethyl acetate for the analysis. Retention indices (RIs) were determined by co-injection of hydrocarbon standards (alkanes C_8_–C_30_) and EO and the active fraction under the aforementioned conditions. The oil components were identified by comparison with data from the literature (The Pherobase, available from http://www.pherobase.com/database/kovats/kovats-index.php), and NIST 05 library profiles, and by co-injection of authentic standards [12], [19].

### Microorganisms

Reference strains of Candida spp. used in this study were obtained from the Netherlands Collection – Central Bureau voor Schimmelcultures (CBS): C. albicans CBS 562, C. tropicalis CBS 94, C. krusei CBS 573, dubliniensis CBS 7987 and Candida rugosa CBS 12.

### Determination of Minimum Inhibitory and Fungicidal Concentration (MIC/MFC)

This is a bio-guided study investigating the antifungal susceptibility of *Candida* spp. to the action of *C. sativum* EO and its active fraction. The MIC test was carried out using 96-well U-bottom tissue culture microplates containing 100 µl/well of RPMI-1640 medium (Sigma-Aldrich, St. Louis, MO, USA). A stock solution of EO emulsion and active fraction was transferred to the first well and serially diluted to obtain concentrations ranging from 1,000 to 0.48 µg/ml. Nystatin (Sigma-Aldrich, St. Louis, MO, USA) and Amphotericin B (União Química Farmacêutica Nacional, SP, Brazil), and 6.25% propylene glycol (vehicle, v/v) were used as positive and negative controls, respectively. Sterility of the culture medium and the EO emulsion, and yeast viability were also checked. Fungal inocula were prepared (530 nm, abs 0.08–0.1) and diluted to reach a final concentration of 2.5×10^3^ CFU/ml in the wells. Plates were incubated at 35°C for 24–48 hrs. MIC was defined as the lowest concentration of the oil that inhibited visible fungal growth [20].

MFC was determined by subculturing an aliquot from each incubated well having a concentration higher than the MIC on Sabouraud Dextrose Agar plates (HIMEDIA Laboratories Pvt. Ltd., Mumbai, India), which were incubated at 35°C for 48–72 hrs. MFC was defined as the lowest concentration of the EO or active fraction that allowed no visible growth on the solid medium [20].

The MFC/MIC ratio was calculated in order to determine if the EO and its active fraction had a fungistatic (MFC/MIC≥4) or fungicidal (MFC/MIC<4) activity [21].

### Mode of Action of the Essential Oil

Tests were performed to determine whether the antifungal effect found is the result of a direct interaction with *Candida* spp. cell wall structure (sorbitol assay) or membrane ionic permeability (ergosterol assay).

#### Sorbitol assay

The MIC of the EO was determined in the presence of sorbitol (osmotic protector) by the microdilution technique [20]. Initially, 100 µL of RPMI-1640 was added to each well of the plate. Subsequently, 100 µl of the EO was transferred to the first well and serially diluted at a ratio of two, to obtain concentrations ranging between 1,000 and 0.48 µg/ml. Finally, 100 µl of inoculum (2.5×10^5 ^CFU/ml) prepared with RPMI-1640 previously supplemented with sorbitol (0.8 M final concentration) (Sigma-Aldrich, St. Louis, MO, USA) was transferred to each well. Yeast growth, media sterility and vehicle were also controlled. Plates were incubated at 35°C and read after 48 hrs and 7 days [22]–[24].

#### Ergosterol assay

To determine whether the EO interacts with ergosterol, MIC was determined against *Candida* spp. by the microdilution method [20] in absence and presence of exogenous ergosterol at concentrations of 100, 200 and 400 µg/ml. Amphotericin B was used as positive control. The vehicle alone served as a negative control and yeast viability in the presence of ergosterol was also checked. Plates were incubated at 35°C for 24 hrs and read afterwards [22]–[24].

### Scanning Electron Microscopy (SEM) Analysis

In order to evaluate the integrity of *Candida* spp. cells using SEM, the biofilms were first developed (2.5×10^5^ CFU/ml) in tissue culture treated chambered glass slides (BD Falcon, Bedford, MA, USA) and treated with the EO (MIC, MFC, MIC times 10, MIC times 20) or with nystatin (MIC). The chambered slides were placed in a shaking incubator (100 rpm) at 35°C for 72 hrs. The samples were then washed twice and kept in glutaraldehyde/PBS 3% (v/v, pH 7.4) for 12 hrs at room temperature. Subsequently, the chambered slides were serially dehydrated with ethanol (50%, 70% and 90%) for 10 minutes and dried for 30 minutes to a critical point. Finally, the chambers were removed and the remaining slides were coated with gold in a metallizer machine and observed using a scanning electron microscope (Jeon JSM 5600LV, Tokyo, Japan) [16].

### Inhibition of Adherence of Candida spp. Biofilms

Adherence tests were carried out using 96-well U-bottom non-treated plates. In order to evaluate the adherence of *Candida* mono-species biofilm, 100 µL of Sabouraud Broth was added to each well and 100 µL of EO emulsion then transferred to the first well and serially diluted at a ratio of two to obtain MIC and sub-MIC concentrations. Finally, 100 µL of yeast inoculum (2.5×10^5^ CFU/ml) prepared with Sabouraud Broth plus sucrose (2%) was added to all wells. The control groups used in this test were: sterility of EO emulsion and culture medium, yeast growth (also used as standard of adherence for comparisons), nystatin (positive control) and vehicle (negative control). Plates were incubated in a shaker (125 rpm) at 35°C for 72 hrs.

#### Quantification of adhered yeast cells

After the incubation period, the liquid contents of all wells were discarded and the plates were washed twice and dried for 45 minutes at room temperature. Then 200 µL of 0.4% crystal violet was added to each well and incubated for 45 minutes. The wells were then washed again and immediately destained with 200 µL of 95% ethanol. After 45 minutes, 100 µL of the destained solution was transferred to the wells of new flat-bottom plates, and crystal violet in each well was measured at 595 nm using a SpectraMax 340 tunable microplate reader (Molecular Devices Ltd, Sunnyvale, CA, USA). The inhibition of adherence was measured indirectly relative to the yeast growth group, which was assigned a value of 100% fungal adherence [adapted from 12, 16, 25].

### Effect of *C. sativum* EO on Proteolytic Activity of *C. albicans*

The strain of *C. albicans* CBS 562 was previously assessed in solid culture medium for its ability to produce proteases. By using circular end of a 8-mm-diameter properly sterilized glass rod, yeasts colonies were picked up from Agar Sabouraud Dextrose plates and *printed* onto Petri plates containing BSA/YNB agar (0.2% bovine-serum albumin, 1.45 g Yeast Nitrogen Base without ammonium sulfate and amino acids, 20 g glucose, 20 g agar *per* liter) at pH 4.0. Plates were incubated at 37°C for 72 hrs [26]. The presence of proteases was detected by the production of a translucent zone around the yeast colony. The level of enzymatic activity, termed the Pz interval, was established as the ratio between the colony diameter (cd) and the colony diameter+translucent zone (cdz), and was classified as: absence of proteolytic activity (Pz = 1.0); positive activity (1.0>Pz≥0.64); or strongly positive activity (Pz<0.64) [27]. Following these procedures, we tested the anti-proteolytic effect of *C. sativum* EO added to the culture medium to be at MFC, MIC and sub-MIC (MIC/2, MIC/4, MIC/8) concentrations. Pepstatin A (5.0 µM) (protease inhibitor) was used as positive control and the vehicle alone was also tested. A control with untreated microorganisms was used as a standard of 100% proteolytic activity.

### Effects of Essential Oil on Human Whole-Genome Expression

We carried out a translational pharmacogenomic study of the effect of *C. sativum* EO and its active fraction on the modulation of human whole-genome expression as a way to study the potential toxic mechanisms on human HeLa cells. *C. albicans* is often found in small amounts in the vagina, cervix, mouth, digestive tract, and on the skin [5]. The HeLa cell line was originally isolated from a human uterine cervix and has already been used extensively in scientific research. HeLa cells can be invaded by *C. albicans* [28] and represent the most widely used human cell line in biomedical research [29]. Our whole-genome expression data can provide a reference tool for past and future experiments by researchers using EO in HeLa cells. These assays were undertaken at the University of Southern California (USC), Ostrow School of Dentistry, Division of Periodontology, Diagnostic Sciences, and Dental Hygiene and Division of Biomedical Sciences (Los Angeles, CA, USA).

Total RNA was isolated from human cells (HeLa CCL-2) treated with the EO or active fraction at IC_30_ (30% Inhibitory Concentration) and subjected to the *Whole-Genome Gene Expression* system using the HumanHT-12 BeadChip V4 (Illumina Inc., San Diego, CA, USA). On each chip, we were able to analyze 34,602 genes using 47,231 probes (representing the human transcriptome) to determine the effect of the naturally-occurring agents on the modulation of gene expression. Bioinformatics analyses were made using the software GeneGo MetaCore (Thomson Reuters, New York, NY, USA), to generate canonical maps and associative networks. This technique has allowed us to determine the specific molecular targets in the human genome that are affected by the EO and its active fraction.

The dataset from our microarray analysis has been deposited in the Gene Expression Omnibus (GEO) platform (http://www.ncbi.nlm.nih.gov/geo), with accession number GSE56431.

### Statistical Analysis

All assays were carried out in triplicate in three independent experiments. An exploratory data analysis was initially performed to determine the most appropriate statistical approach for each assay. Data were statistically analyzed using Graphpad Prism version 5.0 (San Diego, CA, USA). In the test of inhibition of adherence and anti-proteolytic activity, one-way Analysis of Variance and Tukey’s post-test were used, with type I error (α) set as 0.05. Pharmacogenomic data were processed on GeneGo MetaCore software (Thomson Reuters, New York, NY) and DAVID Bioinformatics Resources 6.7 (NIAID/NIH, ML, USA, available from http://david.abcc.ncifcrf.gov/).

## Results

### Essential Oil and Fraction Yields

*C. sativum* EO yield, expressed in relation to dry weight of plant material (%, w/w), was found to be 0.29%, while the active fraction yield expressed as a function of the EO yield (%, w/w) was 7.5%.

### Phytochemical Analysis by GC-MS

The chemical composition of the EO and its active fraction is shown in Tables 1 and 2, respectively. The analyses of their constituents indicated the presence of volatile compounds, mainly mono- and sesquiterpene hydrocarbons. We identified 12 compounds in the EO from *C. sativum*, representing 82.52% of its total composition. Six compounds were identified in the active fraction of the EO, which accounted for 79.52% of the fraction content. The major compounds identified in the EO were decanal (19.09%), trans-2-decenal (17.54%), 2-decen-1-ol (12.33%) and cyclodecane (12.15%), all with similar chemical profiles in the active fraction (Table 1).

**Table 1:** Compounds identified by GC-MS in the essential oil and its active fraction from *Coriandrum sativum* leaves.

R_t_ (min)	R_I_^(a)^	Analytes identified*	Relative Percentage^(b)^	
			EO	Active Fraction
7.62	1004	Octanal	0.34	–
15.00	1198	trans-4-decenal	0.47	–
15.38	1207	Decanal	19.09	4.91
17.74	1263	trans-2-decenal	17.54	24.11
18.15	1272	2-decen-1-ol	12.33	16.57
18.26	1275	Cyclodecane	12.15	16.62
19.61	1307	Undecanal	0.99	–
21.97	1364	2-undecenal	1.17	1.50
22.31	1372	trans-2-undecen-1-ol	0.49	–
23.84	1409	Dodecanal	4.10	–
26.19	1467	cis-2-dodecenal	10.72	15.81
26.44	1473	dodecan-1-ol	3.13	–
26.46	1474	M^(c)^ = 184	–	6.40
31.81	1612	M = 194	0.94	–
34.02	1671	M = 210	11.51	14.09
34.18	1675	M = 194	1.92	–

Notes: ^(a)^ Retention Index; ^(b)^ Percentage fraction of the total area integrated for the chromatogram; (c) M: molecular weight of a non-identified compound. *Only compounds identified or those indicated by their molecular weight are listed.

**Table 2:** Antifungal activity of the essential oil and active fraction of *C. sativum* on *Candida* spp. (values are expressed as µg/ml).

Strain	*C. sativum* EO			Active Fractionfrom *C. sativum* EO			Nystatin			Amphotericin B		
	MIC	MFC	MFC/MIC *ratio*	MIC	MFC	MFC/MIC *ratio*	MIC	MFC	MFC/MIC *ratio*	MIC	MFC	MFC/MIC *ratio*
***C. albicans*** ** CBS 562**	15.6	31.2	2	250	1,000	4	7.8	15.6	2	0.038	0.07	2
***C. tropicalis*** ** CBS 94**	31.2	62.5	2	250	500	2	3.9	7.8	2	0.01	0.02	2
***C. krusei*** ** CBS 573**	15.6	31.2	2	125	250	2	3.9	15.6	4	0.15	0.15	1
***C. dubliniensis*** ** CBS 7987**	31.2	62.5	2	31.2	125	4	3.9	3.9	1	0.002	0.004	2
***C. rugosa*** ** CBS 12**	15.6	31.2	2	62.5	125	2	1.9	15.6	8	0.07	0.61	8

Note: +, fungal growth; ­, no fungal growth.

### Antifungal Activity and Mode of Action of the EO

MIC and MFC values for the EO and active fraction of *C. sativum* and for standard antifungals on *Candida* spp. are illustrated in Table 2. For the EO, MIC values ranged from 15.6 to 31.2 µg/ml, and MFC values ranged from 31.2 to 62.5 µg/ml. The active fraction showed higher MIC and MFC values than the EO, ranging from 31.2 to 250 µg/ml and 125 to 1,000 µg/ml, respectively, for the active fraction group. The vehicle used in the EO emulsion did not affect yeast growth.

The ratio MFC/MIC found for the EO and active fraction demonstrated a fungicidal effect of these samples against the majority of the yeast species tested. As the crude EO was shown to have a better antifungal effect than its active fraction, we decided to perform further antimicrobial assays only with the EO, taking into account the enhanced anti-*Candida* activity of the EO, the costs of the fractionation process, and the potentially higher toxic effects of fractions in relation to their crude oils [12].

Given the promising antifungal effects of the EO, we next studied the mechanism by which the viability of the yeast cells was affected. Our results showed that the antifungal properties of *C. sativum* EO are not related to cell wall biosynthesis pathways, as the findings of the antifungal test were unaltered either in presence or absence of an osmotic protector (sorbitol) (Table 3). Instead, the MIC values of the EO increased between 8 and 16 times with the increase of exogenous ergosterol concentration, indicating that the EO seems to bind to membrane ergosterol and increases ionic permeability, ultimately causing cell death. This same mode of action is observed for amphotericin B, used as positive control (Table 4).

**Table 3:** Effect of *C. sativum* EO on *Candida* spp. cell wall biosynthesis (*sorbitol assay*).

Concentration (µg/ml)	*Candida albicans*(CBS 562)		*Candida tropicalis*(CBS 94)		*Candida krusei*(CBS 573)		*Candida dubliniensis*(CBS 7987)		*Candida rugosa*(CBS 12)	
	Withsorbitol	Withoutsorbitol	Withsorbitol	Withoutsorbitol	Withsorbitol	Withoutsorbitol	Withsorbitol	Withoutsorbitol	Withsorbitol	Withoutsorbitol
**1000**	–	–	–	–	–	–	–	–	–	–
**500**	–	–	–	–	–	–	–	–	–	–
**250**	–	–	–	–	–	–	–	–	–	–
**125**	–	–	–	–	–	–	–	–	–	–
**62.5**	–	–	–	–	–	–	–	–	–	–
**31.2**	–	–	+	+	–	–	+	+	–	–
**15.6**	+	+	+	+	+	+	+	+	+	+
**7.8**	+	+	+	+	+	+	+	+	+	+
**3.9**	+	+	+	+	+	+	+	+	+	+
**1.95**	+	+	+	+	+	+	+	+	+	+
**0.97**	+	+	+	+	+	+	+	+	+	+
**0.48**	+	+	+	+	+	+	+	+	+	+

**Table 4:** Effect of different concentrations of exogenous ergosterol (100–400 µg/ml) on the MIC of *C. sativum* EO and Amphotericin B against *Candida* spp.

Strain	MIC of the EO in absenceof ergosterol	MIC of the EO in presence ofergosterol			MIC of Amphotericin Bin absence of ergosterol	MIC of Amphotericin B in presence of ergosterol		
		100 µg/ml	200 µg/ml	400 µg/ml		100 µg/ml	200 µg/ml	400 µg/ml
***C. albicans*** ** CBS 562**	15.6	500	500	500	0.038	2.4	2.4	4.8
***C. tropicalis*** ** CBS 94**	31.2	250	250	250	0.01	2.4	2.4	9.7
***C. krusei*** ** CBS 573**	15.6	62.5	125	125	0.15	4.8	9.7	9.7
***C. rugosa*** ** CBS 12**	15.6	125	125	125	0.002	4.8	19.5	19.5
***C. dubliniensis*** ** CBS 7987**	31.2	–	–	–	0.07	–	–	–
**Yeast growth in presence of** **ergosterol**	**+**	**+**	**+**	**+**	**+**	**+**	**+**	**+**
**Vehicle**	**+**	**+**	**+**	**+**	**+**	**+**	**+**	**+**

Note: +, fungal growth; ­, no fungal growth or irregular growth due to unclear reasons.

### Scanning Electron Microscopy (SEM) Analysis

**Figure 1:**
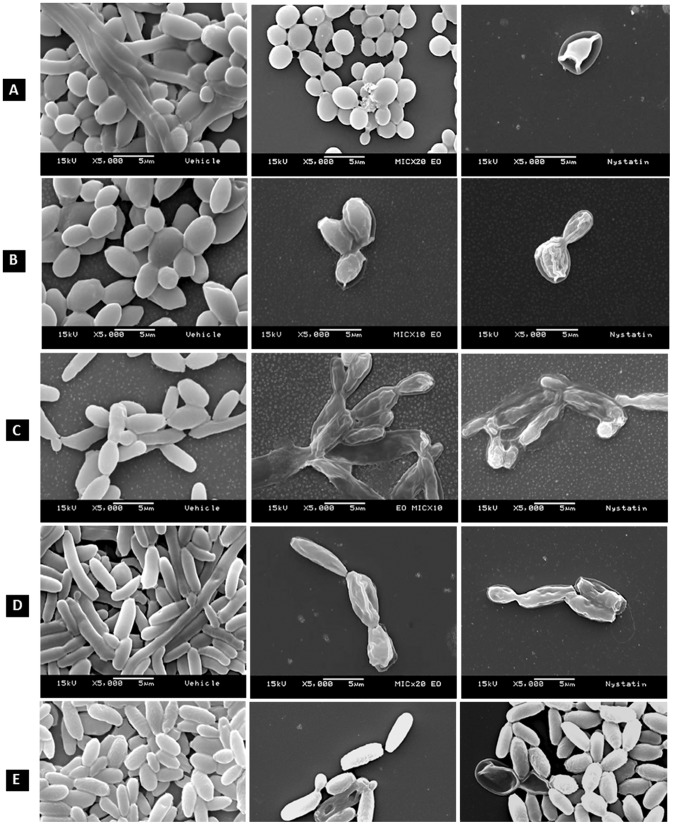
Effects on biofilm morphology. SEM Photomicrographs (5,000x) showing *Candida* biofilm cells after treatment with propylene glycol (vehicle) (left-sided), essential oil from *C. sativum* leaves (middle-sided) and nystatin (right-sided). The most representative cell injuries were caused by the following concentrations of the EO: (A) *C. albicans* – MIC times 20 (312.5 µg/ml); (B) *C. tropicalis* – MIC times 10 (312.5 µg/ml); (C) *C. krusei* – MIC times 10 (156.0 µg/ml); (D) *C. dubliniensis* – MIC times 20 (625 µg/ml); and (E) *C. rugosa* – MIC times 20 (312.5 µg/ml). Nystatin was tested at MIC against all strains (see MIC values in Table 2) and the vehicle did not affect the biofilm integrity.

The effect of *C. sativum* EO on oral *Candida* biofilm integrity was assessed by SEM. Yeast morphology was seen to be disrupted at different concentrations of the EO, with cell damage intensity depending on the species (Figure 1A–E). The most representative injuries for each yeast species and the concentrations of the EO that caused them are as follows: *C. albicans* – MIC times 20 (312.5 µg/ml); *C. tropicalis* – MIC times 10 (312.5 µg/ml); *C. krusei* – MIC times 10 (156.0 µg/ml); *C. dubliniensis* – MIC times 20 (625 µg/ml); and *C. rugosa* – MIC times 20 (312.5 µg/ml). At these concentrations, the EO substantially affected biofilm structure, causing most to turn from turgid to a withered appearance, similar to what was observed for nystatin (positive control) at its MIC. The vehicle did not affect biofilm integrity.

### Inhibition of Adherence of Candida spp. Biofilms

The EO had anti-adherent activity (42–85%) at low concentrations against all strains tested. The best results were found for *C. tropicalis*, on which *C. sativum* EO and nystatin significantly inhibited biofilm adherence at concentrations lower than their MICs: 15.6 and 0.9 µg/ml, respectively, (*P*<0.05) compared to the vehicle which did not affect biofilm adherence (Table 5).

**Table 5:** Inhibitory effects of *C. sativum* EO and nystatin (control) on biofilm adherence of *Candida* spp. after 72 hrs. Values were expressed as mean ± standard deviation of inhibition of biofilm adherence (%).

Concentration (µg/ml)	Inhibition of biofilm adherence (%)									
	*C. albicans*		*C. tropicalis*		*C. krusei*		*C. dubliniensis*		*C. rugosa*	
	EO	Control	EO	Control	EO	Control	EO	Control	EO	Control
0.12	–	–	–	–	–	–	–	–	–	–
0.24	–	–	–	–	7,30±12,65^Abd^	3,93±6,81^Ab^	–	–	–	–
0.48	–	–	–	10,16±5,67^Aa^	7,30±12,64^Abd^	2,24±3,89^Ab^	–	–	–	–
0.97	–	–	–	23,80±20,61^Aa†^	8,05±13,94^Abd^	0,74±1,29^Ab^	–	–	–	–
1.95	–	–	–	49,93±5,95^Ab†^	9,92±17,19^Aad^	16,67±12,64^Aab^	–	2,25±2,88^Aa^	–	–
3.90	–	–	–	84,63±2,65^Ac†^	14,41±12,19^Aad^	0,93±1,62^Aab^	10,18±9,57^Aa^	11,78±7,99^Aa^	–	2,57±4,46^Aa^
7.81	–	–	1,26±2,19^Aa^	88,33±1,36^Bc†^	9,55±8,31^Abd^	3,96±6,81^Ab^	8,23±14,25^Aa^	12,73±11,81^Aa^	–	6,36±5,51^Aa^
15.62	–	–	27,23±26,13^Ab†^	88,96±1,33^Bc†^	1,68±2,02^Abd^	14,79±5,30^Ab^	11,60±12,62^Aa^	26,82±6,57^Aa^	–	10,15±8,81^Aa^
31.25	–	–	67,30±9,35^Ac†^	85,83±3,33^Ac†^	20,78±3,93^Aad^	34,08±18,47^Aa†^	16,46±16,42^Aa^	41,38±4,18^Ba†^	32,57±19,99^Aa†^	16,51±20,39^Aa^
62.50	53,43±8,58^Aa†^	41,92±12,94^Aa†^	85,80±4,32^Ac†^	86,16±3,10^Ac†^	42,13±3,93^Aa†^	46,06±9,74^Aa†^	61,51±3,99^Ab†^	71,81±1,43^Ab†^	68,03±5,56^Aa†^	73,71±4,43^Ab†^
125.00	52,74±7,03^Aa†^	53,26±10,61^Aa†^	89,76±1,69^Ac†^	87,96±1,68^Ac†^	39,51±6,98^Aa†^	40,82±5,39^Aa†^	57,84±6,40^Ab†^	71,46±0,54^Ab†^	47,34±41,04^Aa†^	70,60±5,30^Ab†^
250.00	46,73±5,97^Aa†^	43,04±4,00^Aa†^	85,06±2,44^Ac†^	87,36±0,41^Ac†^	40,63±9,99^Aa†^	41,76±2,53^Aa†^	48,72±4,23^Ab†^	65,42±3,02^Ab†^	48,63±2,40^Aa†^	76,89±2,74^Bb†^

Different uppercase letters in the same line indicate statistically significant difference (p<0.05) between the EO and nystatin at the same concentration against the corresponding strain. Different lowercase letters in the same column indicate statistically significant difference (p<0.05) between the concentrations of the same product (EO or nystatin).

Note: EO: C. sativum essential oil; Control: nystatin; “−” means “no inhibition of adherence”, ^†^Statistically significant difference compared to the vehicle (p<0.05). The vehicle (propylene glycol 6.25%) did not affect the biofilm growth and adherence (p>0.05). Tests were performed in triplicate of three independent experiments and data were submitted to One-way Analysis of Variance (ANOVA) followed by Tukey’s post-test (α = 0.05).

### Effect of C. sativum EO on The proteolytic Activity of C. albicans

**Figure 2:**
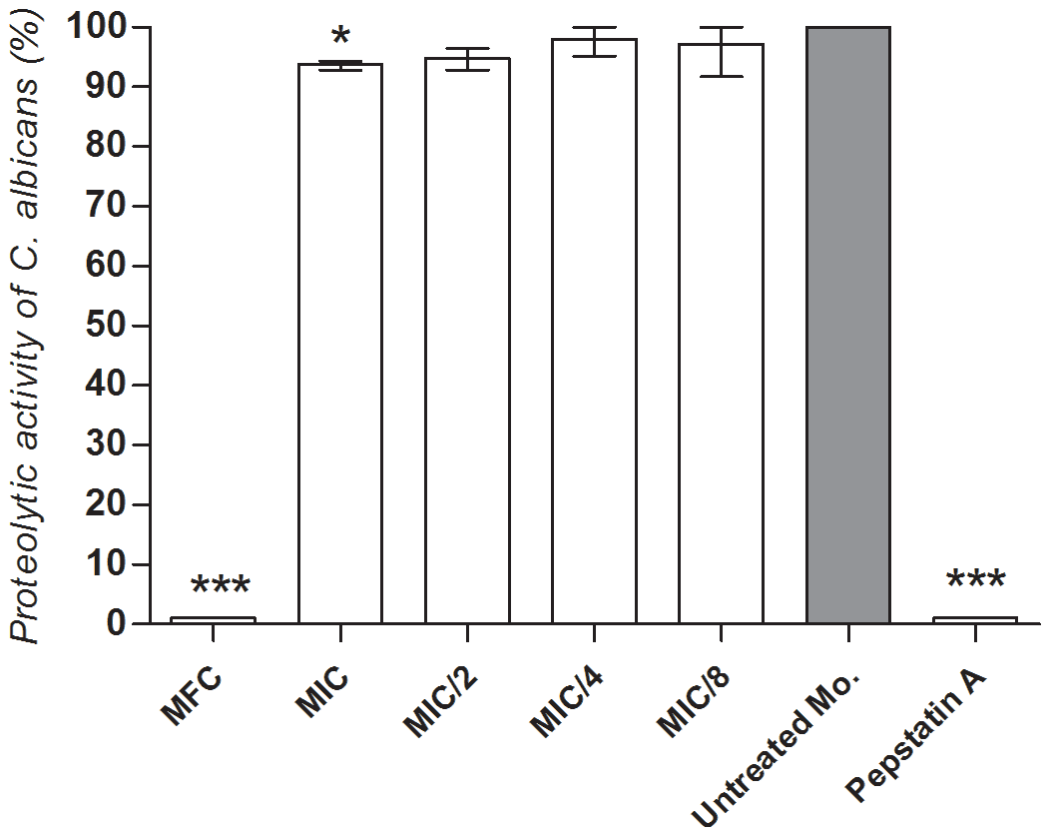
Anti-proteolytic activity. Effect of different concentrations of the EO from *C. sativum* leaves and Pepstatin A (protease inhibitor) on the whole proteolytic activity of *Candida albicans* CBS 562 (One-way ANOVA with Tukey’s post-test, *P<0.05; ***P<0.0001). The group of untreated microorganism was considered as standard of 100% proteolytic activity.

The strain of *C. albicans* CBS 562 had a Pz interval of 0.84, indicating positive activity for proteases. The EO from *C. sativum* leaves decreased the proteolytic activity of *C. albicans*, with a statistically significant difference at MIC compared to the group of untreated microorganisms (100% proteolytic activity) (*P*<0.05), as seen in Figure 2.

### Effects of the Essential Oil on Whole Human Genome Expression

**Figure 3:**
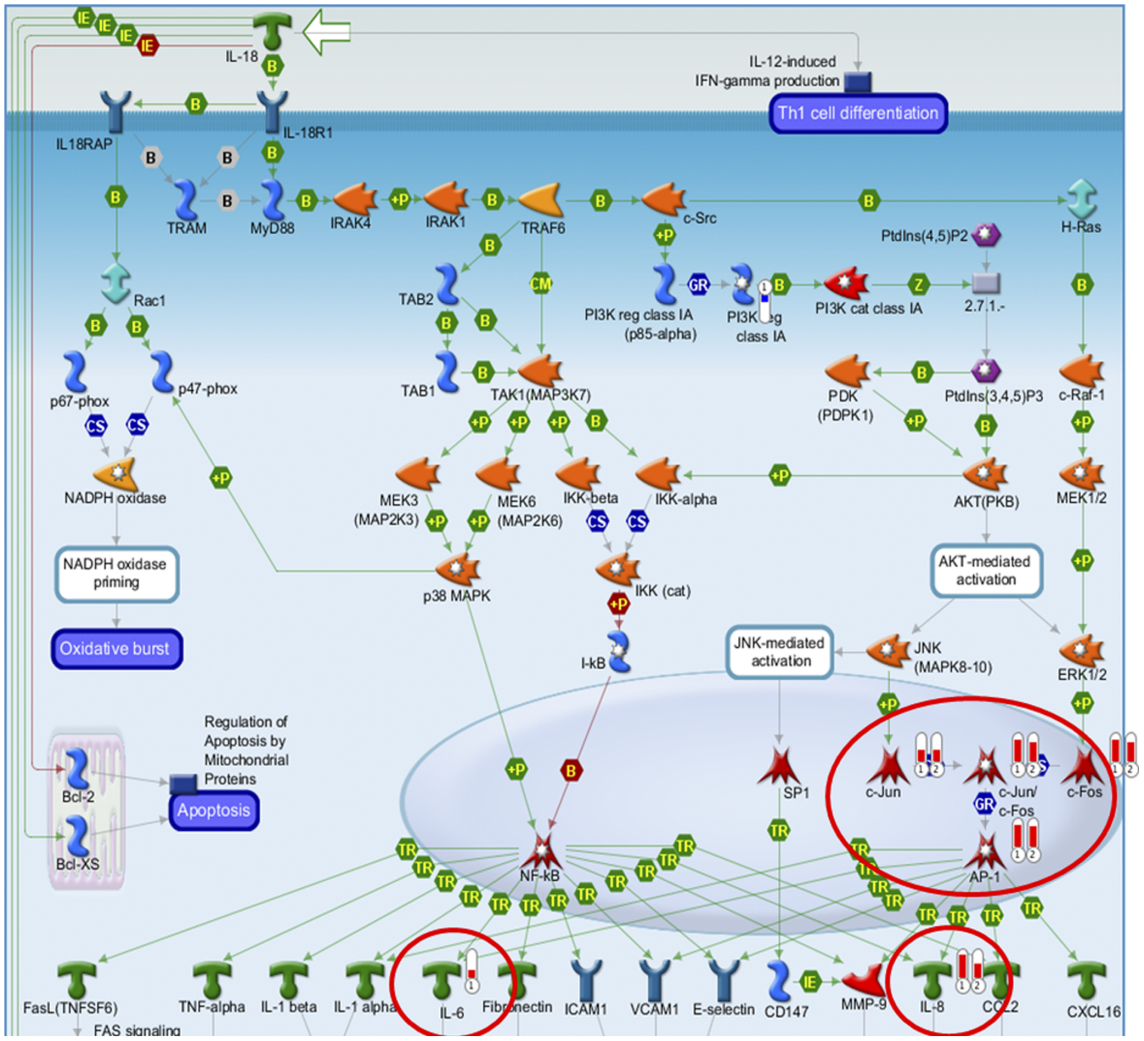
Pharmacogenomic analysis. Representative map of the pathways whose genes involved were up-regulated by the EO (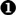) and active fraction (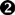) from *C. sativum*. The thermometer next to each component indicates the type (red color means up-regulation) and the magnitude of the modulation of gene expression (MetaCore, Thomson Reuters).

**Figure 4:**
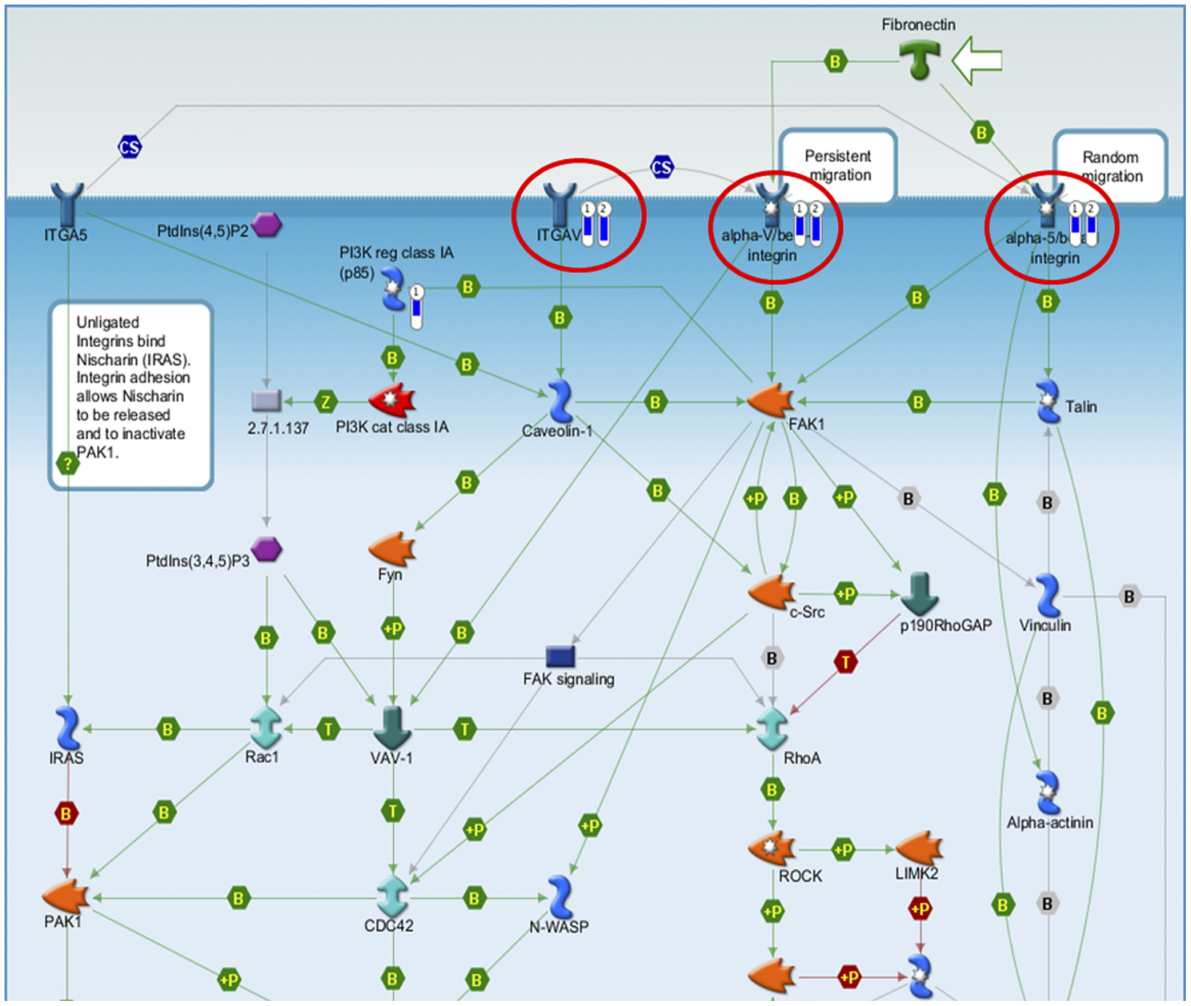
Pharmacogenomic analysis. Representative map of the pathways whose genes involved were down-regulated by the EO (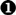) and active fraction (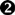) from *C. sativum*. The thermometer next to each component indicates the type (blue color means down-regulation) and the magnitude of the modulation of gene expression (MetaCore, Thomson Reuters).

The EO and active fraction from *C. sativum* L. had low cytotoxicity on human cells with IC_30_ of 359.76 and 366.69 µg/ml, respectively. Gene expression was up-regulated by the EO and active fraction in 109 and 134 genes, respectively, mostly related to chemotaxis (IL-6, IL-8) and proliferation/apoptosis (c-Jun, c-Fos, c-Jun/C-Fos and AP-1). Expression was down-regulated in 28 and 30 genes, respectively, related to 11 adhesion proteins (alpha-4/beta-1-integrin; ITGB1; phosphoinositide-3-kinase; regulatory subunit 2 (beta); alpha-5/beta-1-integrin; alpha-6/beta-1-integrin; alpha-10/beta-1-integrin; alpha-V/beta 3-integrin; alpha-8/beta-1-integrin; integrin-alpha-V (ITGAV); alpha-11/beta-1-integrin; alpha-2/beta 1-integrin), as seen in Figures 3 and 4. The same modulation patterns were found in the DAVID Bioinformatics database (graphs not shown).

## Discussion

Due to recent advances in the exploration of *C. sativum* as a potential therapeutic agent against a number of diseases afflicting humans [10], comprehensive research on its properties has been encouraged. In this study, we investigated the effects of the chemically characterized EO from *C. sativum* leaves on the viability and adherence of *Candida albicans and non-albicans* strains, in both planktonic and biofilm cultures, and evaluated its antifungal mode of action.

Although the amounts may vary, all previous studies seem to agree that the major components of coriander leaf EO are alcohols and aldehydes [18], corroborating our results, as we found decanal, trans-2-decenal, 2-decen-1-ol and cyclodecane as the most prominent compounds. Most of these analytes have also been found as major constituents of coriander leaf samples from Kenya [30], U.S.A. [31], Bangladesh [32], Fiji [33] and Brazil [34]. The mono- and sesquiterpenes found in the leaf EO may be related to the antifungal activity observed. Natural products are considered strong inhibitors of microbial activity when MIC values are lower than 500 µg/ml [35]. Hence, the findings presented herein revealed that the EO from *C. sativum* has strong fungicidal effects against clinically relevant *Candida* species with low MIC and MFC values.

The fractionation process adopted in this bioguided study is well established [12], [36]. However, we found that the EO from *C. sativum* seemed to have a synergism of its constituent compounds, with chemical complexity, since its isolation into fractions led to a decreased antimicrobial activity, as shown by the higher MIC/MFC values found for the active fraction relative to the crude oil. This is in agreement with other studies [12], [16], [37]. It is worth noting that the mixture of numerous molecules in an EO plays a role in defining the fragrance, density, and color of the oil, as well as its cell penetration [38], lipophilic or hydrophilic attraction and fixation on cell membranes, and cellular distribution. Thus, for biological purposes, it is more informative to study the crude oil rather than some of its individual components because of the importance of the concept of synergism [39]. Allied to this fact are the poorer results observed in this study for the active fraction of *C. sativum* EO in comparison to the unfractionated oil, which led to our selection of the EO for further microbiological testing.

The antifungal activity of EO’s depends fundamentally on their ability to pass through the cell wall and penetrate between fatty acid chains of the lipid bilayer, altering membrane fluidity and permeability and damaging membrane proteins, leading to degradation of the cytoplasmic membrane and to cell death. Loss of cell homeostasis, leakage of cell contents, and lysis are the critical consequences of these induced alterations in membrane structure and function [39], [40]. Although the antifungal activity of *C. sativum* leaf EO was known, its modes of action on the viability and pathogenicity of yeasts remained poorly studied. Herein, we investigated two possible chief mechanisms for *C. sativum* leaf EO antifungal effects on *Candida* yeasts: (i) blocking cell wall biosynthesis or (ii) increasing membrane ionic permeability. Our findings showed that the EO seems to bind to membrane ergosterol, rendering the cell membrane more permeable and resulting, ultimately, in cell death, as ergosterol is crucial to maintain cell integrity, viability, function and normal growth [41]. This same mechanism accounts for the antifungal activity of polyenes such as nystatin and amphotericin B [42]. Tests of the effect of *C. sativum* EO on ergosterol biosynthesis (e.g. enzyme-targeting and analysis of 2-^13^C-acetate incorporation) [43] were not undertaken in our study, but are strongly recommended as other cell targets might also be involved in this EO antifungal activity.

Although it has a rather different chemical composition, the EO from *C. sativum* seeds also has antimicrobial activity, whose primary mode of action is cell permeabilization and consequent DNA leakage in yeasts [9] and bacteria [44] based on flow cytometry analyses.

The study of antifungals’ mode of action is an important strategy for limiting the emergence of resistance to the currently available agents, as well as for developing safer and more potent drugs against fungal infections [45]. In addition to the primary mechanisms of action of antifungals (β-Glucans biosynthesis blocking and disturbance in ergosterol synthesis or function) [42], some agents can also have alternative effects on other targets, for instance on mitochondrial activity, which plays a role in generation and regulation of Reactive Oxygen Species (ROS), calcium homeostasis and ATP production, all related to cell viability [46]. Given this, we suggest the study of *C. sativum* EO action upon other strategic targets associated with the regulation of metabolic processes in the fungal cell, in order to have its antifungal potential comprehensively elucidated.

From a clinical perspective, fungal biofilm formation leads to negative consequences to health, since such biofilms act as protected reservoirs of microorganisms [47] displaying properties that are extremely different from planktonic populations, mainly high-level resistance to a number of antifungal agents [48]. An association has been demonstrated between biofilm formation and a pattern of increased virulence and resistance in *Candida* species [48], [49]. Hence, the study of the antifungal activity of natural products should prioritize biofilm growth models to reach greater reliability and closer approach to medically-relevant conditions. In our study, we used electron microscopy to assess the effect of *C. sativum* EO on oral *Candida* biofilm integrity. We demonstrated that the EO at relatively low concentrations (156.0 to 312.50 µg/ml) clearly disrupted yeast morphology. These findings are consistent with the mode of action, as we observed that the cell membrane structure seemed to be altered to some extent but not the cell wall, indicating that the EO possibly acts on membrane permeability rather than on cell wall biosynthesis. As is the case with gram-positive oral bacteria, simple conformational change in *Candida* biofilms caused by the action of the EO could render the yeast cells more susceptible and less virulent [50].

The initial stages of biofilm formation and adherence to a substrate material are mediated by both abiotic factors, such as surface hydrophobicity, and biotic factors, such as increased expression of adhesins and other cell-surface proteins [51]. Hence, the inhibition of adherence of yeast cells may constitute a promising target for disrupting the initial stages of biofilm formation in *Candida* spp. [52]. In this study, *C. sativum* EO was checked for its ability to prevent adherence of 72-hour *Candida* biofilms onto a polystyrene substrate. We demonstrated that *C. sativum* EO accounted for a statistically significant 42–85% inhibition of biofilm adherence at a concentration of 62.2 µg/ml on the tested strains. In another study, anti-adherent activity was also found for the active fraction from *C. sativum* EO against *Streptococcus mutans*. Using the same method presented herein, the authors demonstrated that the selected fraction inhibited 95% of biofilm adherence at a concentration of 31.2 µg/ml [12].

The effect of *C. sativum* EO on oral *C. albicans* biofilm development kinetics has been previously established. A clear effect of the oil at 125 µg/ml on biofilm formation was observed after 48 hrs, characterized by an increase in the lag phase and a decrease in the biofilm growth [16].

Following adhesion to host surfaces and colonization, *C. albicans* hyphae can secrete proteases, which have been found to facilitate active penetration into the host cells [53]. In addition, secreted proteases are thought to favor extracellular nutrient acquisition [54]. New therapy approaches involving the inhibition of secreted hydrolases have been developed for the control of oral candidiasis, including the use of medicinal plants [55]. The inhibitory effect of *C. sativum* EO on aspartic proteases secreted by *C. albicans* was evaluated using the agar diffusion method. Despite shortcomings related to sample liposolubility-dependent diffusion and lack of specificity, this technique has been reported by a number of studies to provide preliminary inhibition rates of the whole-proteolytic activity of microorganisms [56]–[58]. We found that *C. sativum* EO at MIC caused a statistically significant decrease of about 7–8% in the proteolytic activity of *C. albicans*. These findings suggest that the EO can act by interfering with yeast invasion mechanisms, which could prevent the development of candidiasis. As there is no current study in the literature investigating this property for *C. sativum*, determination of extracellular proteinase activity in the presence of *C. sativum* EO using spectrophotometric tests should be undertaken.

Even though most EO are likely to be devoid of carcinogenicity – but not of cytotoxicity – some of them, or rather some of their compounds, may become secondary carcinogens after metabolic activation [39], [59]. It is therefore essential to carry out toxicological assays of any plant-derived material to be used in humans as a herbal formulation. In our study, we performed a preliminary pharmacogenomic analysis of the EO and active fraction from *C. sativum,* to establish how these samples would interfere with the human genome expression. Both the EO and its active fraction had relatively low cytotoxic activity and the putative mechanisms we identified involve the pathways of pro-inflammatory chemokines (e.g., IL-6, IL-8) and the mitogen-activated protein kinase pathway (i.e., proliferation and apoptosis processes), as well as adhesion proteins (mostly integrins). This is the first translational study demonstrating the pharmacogenomic profile of *C. sativum* EO in the international literature. As such, our findings provide genomic information for further *in vitro* and *in vivo* research.

*C. sativum* is approved for food uses by the Food and Drug Administration, which granted it the GRAS (Generally Regarded as Safe) status, and also by the Council of Europe (CoE) [58]. Based on the history of consumption of *C. sativum* leaves without reported adverse effects, and lack of toxicity of the major constituent from the seeds EO (linalool) [9], the use of *C. sativum* as an additional food ingredient is considered safe [60]. The median lethal dose (LD_50_) of *C. sativum* seeds EO was determined as 2.257 ml/kg [61]. Nevertheless, the LD_50_ of the leaves EO has not been reported in the literature and remains unclear, as chemical composition of the EO from the leaves differs substantially from that of the seeds.

As discussed earlier, several other applications of EO are possible in medical devices. Hence, the use of this particular EO should be further investigated with regard to prevention of *Candida* infections in clinical settings [9].

## Conclusions

The EO from *C. sativum* leaves has strong antifungal and anti-adherent activity against *Candida* spp., as well as anti-proteolytic activity against *C. albicans*, and acts by increasing cell membrane ionic permeability rather than disturbing cell wall biosynthesis. All these properties are probably due to the synergistic effect of the constituent mono- and sesquiterpene hydrocarbons identified. Furthermore, pharmacogenomic analyses revealed that *C. sativum* EO has relatively low cytotoxicity, with putative mechanisms through modulation of gene expression in chemokine and mitogen-activated protein kinase pathways (proliferation/apoptosis), as well as expression of adhesion proteins.

Other toxicological bioassays and phase I and II clinical trials are now needed to further investigate the promising antifungal activity of *C. sativum* EO as a potential candidate in the treatment of oral diseases, such as denture-related candidiasis.
